# Foreign Body in the Air Passages—Retained Fifteen Months

**Published:** 1854-03

**Authors:** G. R. Henry

**Affiliations:** Burlington, Iowa


FOREIGN BODY IN THE AIR PASSAGES—RETAINED FIFTEEN MONTHS. —By G. R. HENRY, M. D., Burlington, Iowa.—Sept. 22, 1852, Mr. J. L., of Yellow Springs in this county, brought to me his child three years old, who had about two weeks previously suffered a water-melon seed to pass into the larynx; I was unable to detect the presence of any foreign substance, and consequently declined performing the operation of tracheotomy, for which purpose the child had been brought to me. The child was suffering from inflammation of the left lung, and I felt satisfied from repeated examinations that if the seed was yet in the passages, that it was so impacted into one of the smaller bronchial tubes of the left lung that an operation would be unavailing. Giving this as my opinion, the father took the child home, to the charge of Dr. F. whose patient he was, and under whose care she rapidly recovered from the attack of pneumonia and regained her usual health, but would occasionally be troubled with severe attacks of coughing. I have not seen this little patient since she was first brought to me. But Mr. L. called a few days ago at my office, and said that on the 13th of December 1853, during a violent attack of coughing she had thrown up the water-melon seed which had been forced into the trachea fifteen months previously; the seed being scarcely altered from its original condition; since which time the child has been free from cough.

This case shows the remarkable power that nature possesses, of adapting herself to the exigencies of different situations, and teaches us that persons beyond the reach of art may yet be cured by the *vis medicotrix naturæ.*

Had the child been brought to me immediately after the accident occurred, I would have operated with much hope of success, for then the foreign body was loose, and as her father expressed it, "would flip" when she breathed, but when I saw her there was no *positive* evidence that the seed had been coughed up, and there was certainly great danger of increasing the inflammation which had already supervened.—*Iowa Med. Journal.*

